# Cortical Axons, Isolated in Channels, Display Activity-Dependent Signal Modulation as a Result of Targeted Stimulation

**DOI:** 10.3389/fnins.2016.00083

**Published:** 2016-03-07

**Authors:** Marta K. Lewandowska, Miloš Radivojević, David Jäckel, Jan Müller, Andreas R. Hierlemann

**Affiliations:** Bio Engineering Laboratory, Department of Biosystems Science and Engineering, ETH ZürichBasel, Switzerland

**Keywords:** microelectrode array, axon, axonal propagation, neuronal stimulation, axonal channels, electrophysiology

## Abstract

Mammalian cortical axons are extremely thin processes that are difficult to study as a result of their small diameter: they are too narrow to patch while intact, and super-resolution microscopy is needed to resolve single axons. We present a method for studying axonal physiology by pairing a high-density microelectrode array with a microfluidic axonal isolation device, and use it to study activity-dependent modulation of axonal signal propagation evoked by stimulation near the soma. Up to three axonal branches from a single neuron, isolated in different channels, were recorded from simultaneously using 10–20 electrodes per channel. The axonal channels amplified spikes such that propagations of individual signals along tens of electrodes could easily be discerned with high signal to noise. Stimulation from 10 up to 160 Hz demonstrated similar qualitative results from all of the cells studied: extracellular action potential characteristics changed drastically in response to stimulation. Spike height decreased, spike width increased, and latency increased, as a result of reduced propagation velocity, as the number of stimulations and the stimulation frequencies increased. Quantitatively, the strength of these changes manifested itself differently in cells at different frequencies of stimulation. Some cells' signal fidelity fell to 80% already at 10 Hz, while others maintained 80% signal fidelity at 80 Hz. Differences in modulation by axonal branches of the same cell were also seen for different stimulation frequencies, starting at 10 Hz. Potassium ion concentration changes altered the behavior of the cells causing propagation failures at lower concentrations and improving signal fidelity at higher concentrations.

## Introduction

The axon is an important element in cell-to-cell communication, since it connects a neuronal cell body with its numerous post-synaptic partners. Many cells have a single axon whose initial segment is the site of action potential generation (Coombs et al., 1957; Fatt, 1957; Fuortes et al., 1957; Häusser et al., 1995), and which often grows many separate branches that then propagate the action potential (Sasaki et al., 2012a). As such, a good understanding of axonal behavior and capabilities is necessary not only to increase our understanding of the nervous system, but also because of its potential dysfunction as a result of accident and its potential for regeneration (Fawcett, 1992). Although, classical experiments in invertebrates and peripheral nerves used axons as their model systems (Huxley and Stämpeli, 1949; Hodgkin and Huxley, 1952), the axons of the mammalian central nervous system, particularly those that are unmyelinated, have remained largely inaccessible to current methods because they are so small (Kress and Mennerick, 2009).

A number of researchers have recently put into question the notion that axons merely act as cables, reliably transmitting action potentials along their lengths (Debanne, 2004; Debanne and Boudkkazi, 2010), and have developed some techniques with which to read out signals from axons (Kress and Mennerick, 2009). Dentate gyrus granule cells are known for their large mossy fiber boutons, whose size is around 3–5 μm, enabling patch clamping of these structures (Bischofberger et al., 2006). Activity-dependent broadening was examined using this technique, and voltage-gated K^+^ channels were found to be the molecular mechanism responsible for the observed behavior (Geiger and Jonas, 2000). Na^+^ channels were also studied with this technique, and found to have different functionality with regard to action potential propagation depending on whether they were in the bouton (amplification) or in the axon (reliability and speed; Engel and Jonas, 2005). A recently developed technique, the patching of axonal blebs, swellings resulting from slicing of neuronal tissue, has made possible axonal recording from axons without patchable boutons (Shu et al., 2006). Kole used double and triple patch clamp techniques to patch the soma, axon initial segment (AIS), and an axonal bleb of cortical layer V cells, and examined ion channel distributions in the AIS. They studied K^+^ channels, and found that Kv1 channels shape the action potential, regulate neurotransmitter release, and are able to integrate subthreshold activity (Kole et al., 2007). Sasaki targeted axons of CA3 pyramidal cells, and studied action potential broadening via local application of glutamate and adenosine A1 receptor antagonists. They found that “depolarization-induced K^+^ channel inactivation is likely to underlie the AP broadening” (Sasaki et al., 2011). Finally, the older technique of extracellular recording using cell-attached configurations has been given new life via fluorophore-coated pipettes, allowing both axon, and patch pipette to be visible under fluorescent light (Ishikawa et al., 2010; Sasaki et al., 2012b). Sasaki found that axonal geometry modulated somatic influence differently to proximal and distal synapses, i.e., the observed action potential broadening changed with distance from the soma (Sasaki et al., 2012a).

While extracellular techniques are not a new approach, the aggregation of many electrodes into a microelectrode array whose geometry is capable of studying axons is quite new (Dworak and Wheeler, 2009). This is an approach that uses, not one, but from tens to thousands of electrodes to read out signals from single cells and even subcellular structures, such as axons, and to selectively stimulate them. Two different approaches utilizing transistor arrays include Si nanowire field effect transistors with contact areas as small as 0.06 μm^2^, which require external readout electronics (Wu et al., 2004), that were used to stimulate and read out from multiple parts of the same neuron (Qing et al., 2010), and a complementary-metal-oxide-semiconductor (CMOS) integrated multi-transistor array featuring 16,384 sensors with a resolution of 7.4 μm (Lambacher et al., 2011) that was used to study propagation along retinal ganglion axons (Zeck et al., 2011). CMOS microelectrode arrays (MEAs) that have been realized include an active pixel sensor which can read out all 4096 electrodes simultaneously (Imfeld et al., 2008), and which was used to study cardiomyocytes and burst dynamics of neurons (Berdondini et al., 2009) as well as an 11,011 electrode array (Frey et al., 2010) which has been used to study brain slices (Frey et al., 2009), retinal ganglion cells (Fiscella et al., 2015), and dissociated neurons. Bakkum et al. used this latter MEA to trace axonal paths, including multiple branches, over hundreds of electrodes, and observed differences in velocity over axonal arbors and observed that “many-fold velocity differences exist locally within a single neocortical axon.” Because the axonal signals that they observed were small, seven to 25 trials had to be averaged in order to yield a detectable signal, exceeding a 5-sigma threshold. (Bakkum et al., 2013) This problem was overcome in recently published articles: by adding a simple microfluidic device on top of the microelectrode array featuring thin and narrow channels into which only the axons could grow (Taylor et al., 2003, 2005), and which amplified axonal signals such that single propagations could be detected above the noise (FitzGerald et al., 2008; Pan et al., 2013; Lewandowska et al., 2015). The idea for this technique, which was pioneered almost 40 years ago (Campenot, 1977), is still used in its original form (Campenot, 2009) and has been enriched by microfabrication techniques. Using spontaneous spiking, Shimba et al. detected velocity changes along axons resulting from high frequency bursts (Shimba et al., 2015). Lewandowska et al. were able to stimulate near the cell body as well as along the axon in the channel, and found that complex signals in channels could be traced back to individual cells, as well as that signal to noise in the axonal channels increased over time (Lewandowska et al., 2015).

In the present work we demonstrate how our approach, using a high-density microelectrode array (HD-MEA) with an axonal isolation microchannel device on top (Lewandowska et al., 2015), can be used to study axonal modulation of orthodromic action potentials in very thin branches of mammalian cortical axons. The HD-MEA, fabricated in complementary metal-oxide-semiconductor (CMOS) technology, features 11,011 Pt electrodes with a center-to-center pitch of 17.8 um, thus enabling sub-cellular resolution (Frey et al., 2009, 2010). The poly(dimethylsiloxane) (PDMS) axonal isolation device was made of two cell culture chambers connected by long and narrow microchannels into which axons grew, and which amplified axonal signals (Lewandowska et al., 2015). This ensemble of micro-technologies is a powerful tool for studying axonal information processing. Single cells and their processes could be identified based on spontaneous spiking, and the respective somata were then stimulated at moderate to high frequencies while signals from their branching axons were recorded with high fidelity. Because of the large number of readout electrodes and their non-invasive nature, recordings of multiple axons in multiple places could be made without damaging the cells. Extracellular spike shapes were observed to vary dramatically as a result of stimulation: waveforms became wider by as much as a factor of three, peak height decreased down to 20% of the original height, and propagation velocity slowed down to 15% of the original velocity. Moreover, cells reacted to stimulation at different frequencies in unique ways, which corresponded to their spontaneous behavior: faster spiking cells could propagate higher stimulation frequencies. Additionally, we observed axonal branches of the same cell modulating action potentials differently even at low (10 Hz) stimulation frequencies.

## Methods

### Microelectrode array chip

The design, fabrication, and characterization of the high-density microelectrode array can be found elsewhere (Frey et al., 2010). Relevant features of the chip are the following: a 1.75 × 2.0 mm^2^ array of 11,011 bright Pt electrodes with 6 × 8 μm^2^ area and 17.8 μm pitch resulting in an electrode density of 3150 electrodes per mm^2^. Around the periphery of the array, on the same complementary metal-oxide-semiconductor (CMOS) chip, are: three-stage amplification, signal conditioning, analog-to-digital conversion, and stimulation buffers. A switch-matrix integrated into the electrode array allows an almost arbitrary subset of 126 electrodes to be read out simultaneously, and rerouted within 1.4 ms (Frey et al., 2009, 2010). Each of the electrodes can be routed to one of 32 stimulation buffers controlled by two independent waveform generators. The stimulation buffers enable voltage or current stimulation of any electrode on the array.

### Axonal isolation device

Fabrication and first measurements with the axonal isolation device are described in our earlier work (Lewandowska et al., 2015). Briefly, molds for PDMS were made using soft lithography and a three layer SU-8 process on silicon wafers: one layer of SU-8 3005 for the axonal channels and two layers of SU-8 100 for the culture chambers. SU-8 3005 was spun at 5000 rpm for 60 s to fabricate 5 μm high channels. Fabrication was completed, including development and hard baking before constructing culture chambers. SU-8 100 was spun at 1000 rpm for 30 s, soft baked, and, then, a second identical layer was spun on to create the 0.7 mm high culture chambers. Such high structures allowed us to pour a layer of PDMS over the wafer, which resulted in culture chambers that were open from both sides. The resulting PDMS piece featured two culture chambers that were pill-shaped, each with an open area of 2.5 × 1.0 mm^2^, and the axonal channels in between them were 5–8 μm wide, 5 μm high, and 500 μm long. It is shown in Supplementary Figure 1.

The CMOS chip was glued to a custom printed circuit board and wire-bonded to the PCB. The thin (~0.5 mm) layer of PDMS was cut into pieces smaller than the CMOS chip, but slightly larger than the electrode array and placed onto the chip with a pair of tweezers under a stereomicroscope. The open wires and other electrical openings were then protected with a layer of PDMS.

### Neuronal cell plating

Permission to perform animal experiments was granted under animal license 2358, approved by the Basel-City Cantonal Veterinary Authority. Primary neurons were isolated from embryonic day 18 (E18) Wistar rats in accordance with Swiss federal laws on animal welfare. Timed pregnant females obtained from Charles River Laboratories, France, were anaesthetised with isofluorane. The female was immediately sacrificed with a guillotine, and embryos were extracted. Embryos were sacrificed by severing the spinal cord. Their primary cortices were removed and placed into Hank's Balanced Salt Solution free from Mg^2+^ and Ca^2+^.

Cortices were dissociated chemically in 0.25% trypsin with ethylene-diamine-tetra-acetate (EDTA) for 15 min at 37°C. The trypsin was washed away, and then tissue was mechanically dissociated by trituration using a pipette tip. Cells were filtered to exclude clumps of tissue, and the single cells were counted. Typical plating densities were 10 to 20k cells per culture chamber or 1000–2000 cells per mm^2^. Cells were kept in Neurobasal-based plating medium (Neurobasal supplemented with horse serum from HyClone, GlutaMAX, and B27) for 24 h, and then, medium was replaced with Dulbecco's Modified Eagle Medium (DMEM)-based medium (DMEM supplemented with horse serum, GlutaMAX, and sodium pyruvate). Medium was changed once a week. Experiments were conducted in a humidified incubator (RH 65%) at 36°C and 5% CO_2_. Chemicals were purchased from Invitrogen unless otherwise noted.

### Electrophysiological recordings

#### Spontaneous spiking readout protocols

Candidate somata were located on the chip based on their spontaneous spiking profiles and their spike shapes. Since, there are many more electrodes than readout channels (see Methods Section Microelectrode Array Chip), so that not all electrodes can be read out simultaneously, many different electrode configurations were used sequentially in order to acquire data over the whole chip. The chip area was thus sampled in an unbiased way by first scanning for spontaneous activity with 100 random configurations, each lasting 60 s. Electrodes, which recorded large negative-first spikes were identified as being located close to putative somata. We added the criterion that their spiking rates between 2 and 20 Hz (excluding rarely and tonically spiking cells) to aid in cell identification. These spontaneous scans were performed on seven different chips.

Cell somata and their axonal branches, what we will call the “footprint” of the cell, were located on the microelectrode array using spontaneous spiking. Eight to twelve isolated electrodes, that were close to 8 to 12 different putative somata based on the previous step, were chosen per chip. For each of these selected electrodes, two additional adjacent electrodes were selected, so that each putative neuronal unit had three electrodes assigned to it. These 24 (8 × 3) to 36 (12 × 3) electrodes were fixed (they did not change) over all subsequent recording configurations. The other “non-fixed” electrodes were chosen randomly, as before, so that the entire chip space could be sampled, again for 30–60 s per configuration. Usually 110–130 configurations were needed to record from all of the electrodes on the chip. Each three-electrode group (putative soma) was then individually spike-sorted.

Creating a triggered spontaneous profile of a cell required spike sorting, and this was done using custom scripts in Matlab, and UltraMegaSort2000 (Hill et al., 2011). Each three-electrode group was sorted individually. Signals were filtered in both forward and reverse directions with a Butterworth filter. Then, threshold detection was performed (the chosen threshold was six times the standard deviation of the noise), and spikes were cut out and realigned. Small “mini-clusters” were used to compute *k*-means, and then mini-clusters were combined based on energy calculations. Sorted spikes were then visually inspected, and several smaller clusters were combined manually into much bigger clusters, which made up the overwhelming majority of spikes detected on the electrodes of interest. Signals on all other electrodes of the array within a 6 to 10 ms time window were then cut out according to spike times on the electrodes of interest to yield a spontaneous spiking profile for each neuron. Performing such an analysis required combining data from many different configurations, each recorded for a short time period (30–60 s) over the course of 1.5–2 h. As a result, the number of spikes on adjacent electrodes varied substantially. Such an approach, however, provided an unbiased sample of the average behavior of the neuron, and could be used to create single configurations that sampled many parts of the same cell simultaneously. The resulting data is what we call a triggered spontaneous scan over the chip, where the trigger is the spiking of a cell of interest. The electrical profile of the neuron over the chip is what we refer to as its footprint.

#### Stimulation protocols

Medium to high frequency stimulation near the soma was used to stimulate cells, and the responses were recorded from the axonal branches. Stimulation was elicited via biphasic (positive then negative) voltage pulses that were precisely timed and delivered from one of the on-chip stimulation buffers. Voltage pulses are naturally charge-balanced, as they produce opposite-sign currents at their onset and end. The stimulation electrode was chosen by stimulating the electrodes around the putative soma and observing the response in the channels. Sometimes only one electrode was able to evoke an action potential, and at other times, several electrodes evoked an action potential, but generally there was one electrode that was more effective in exciting a specific neuron at a lower voltage than the others. This electrode was used for all later stimulations of that given cell. The voltage used was always the lowest voltage needed to evoke the cell with 100% efficacy plus 50 mV, generally around 500–800 mV (inside the water window for all media used in this study), using bright Pt electrodes. Each phase of the voltage pulse was 200 μs long. Recording was performed during and immediately following the stimulation.

Each stimulation protocol was composed of a short stimulation period followed by a long rest period. Generally 3000 to 4000 stimulations were performed at a given frequency, and then the cell was allowed to rest for 30 min before the next stimulation sequence was applied.

Three different types of medium were used when performing stimulations. The “normal” artificial cerebral spinal fluid (aCSF), which very closely resembled our normal culture medium, was used for most experiments and it contained (in mM) 1.5 CaCl_2_, 0.75 MgSO_4_, 5 KCl, 40 NaHCO_3_, 100 NaCl, 0.8 NaHPO_4_, 1 sodium pyruvate, 25 D-glucose, and 0.5 GlutaMAX. Low [K^+^] aCSF contained all of the same ingredients, except 2 KCl, and 103 NaCl to osmotically balance the solution, while high [K^+^] aCSF contained 8 KCl, and 97 NaCl to compensate. Cells were kept in DMEM-based medium, which was changed to aCSF 20 min before beginning an experiment.

### Data analysis

All data was analyzed in Matlab using custom software. Spike detection during high-frequency stimulation was performed using template matching. Templates were made for the spike on each electrode by creating a median average of 30 traces at a low stimulation frequency. This template was then convolved with the data to resolve the spike. Timing information was extracted by finding the minimum of the first derivative in the data around the spike that had been found.

## Results

### Axons in channels could be correlated to somata based on spontaneous spiking

**Figure 1:**
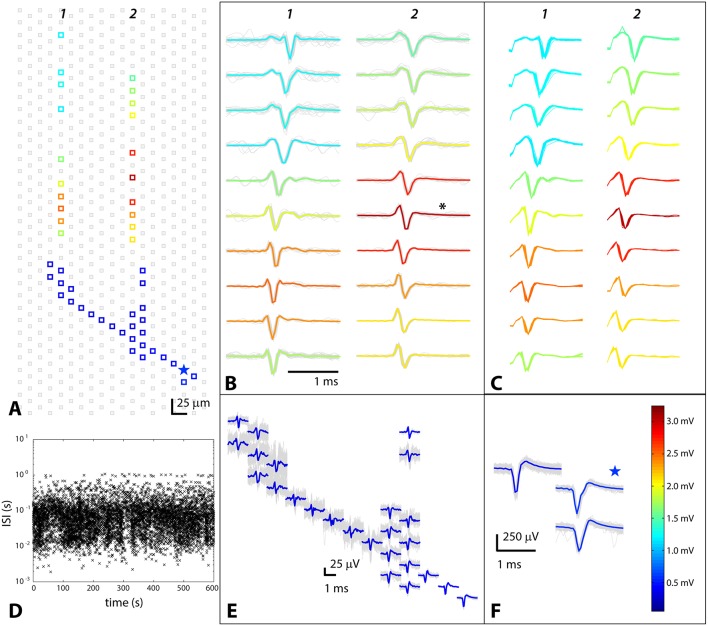
**Triggered spontaneous scan to identify axon and axonal branches belonging to a cell whose somatic location was known**. Data from electrodes near the soma were spike sorted, and the resulting spike times were correlated with spikes on other electrodes on the chip. The colors of the spikes encode their amplitude, as indicated by the scale bar in **(F)**. **(A)** Positions of electrodes where spikes were detected, with putative soma designated by a star; **(B)** Spontaneous axonal waveforms in PDMS channels; asterisk denotes electrode from which data shown in Figure 2 was taken; **(C)** Stimulation-evoked spikes on the same electrodes as in **(B)**; **(D)** Inter-spike interval (ISI) vs. time to illustrate spontaneous behavior of the cell; **(E)** Small axonal spikes outside of PDMS channels buried in noise; **(F)** Spikes on fixed electrodes near soma; star denotes putative soma location.

The high density of electrodes paired with the amplification of axonal signals by the PDMS axon channel device made it possible to identify neurons, including their axonal arbors inside of the channels. Somata and axonal branches were identified using triggered spontaneous scans over a single chip (see Methods Section Spontaneous Spiking Readout Protocols). Spikes on fixed electrodes, that were associated with a putative soma, were spike sorted, and the times were correlated with spikes over the rest of the array to create a profile of a single cell, termed a “footprint.” The number of electrodes monitoring an axon depended on the size and extent of the axonal arbor and ranged from 40 to 90. These electrodes were chosen based on the spontaneous spiking “footprint.” One such cell identification is shown in Figure 1. Spontaneous spikes on the designated electrodes are shown, as are the electrodes on which the spikes were recorded. All of the spikes recorded on the non-fixed electrodes are shown and their mean traces are overlaid in color. In some cases, as Figures 1A,E show, it was also possible to identify parts of axonal arbors outside of channels, but the spikes on those electrodes were always smaller than at the soma and much smaller than in the channels. Averaging many signals together enabled identifying the path along which the axon grew, but single traces were usually lost in the noise. Figure 1E clearly shows that while average footprints of the axon could be extracted, the actual single traces were small and noisy. In contrast, in the channels signals were significantly amplified, even with peak-to-peak heights around 3 mV, as the scale bar in Figure 1F shows (scale bar and color coding applies to all panels of Figure 1). This very large amplification was not typical, but signal amplification by at least a factor of 2 was always seen. The signal to noise in the axon channels ranged from 10 to 300.

### Stimulating cells identified via spontaneous spiking yielded the same spike shape and timing

Our goal was to first identify cells whose axons had grown into channels and then to stimulate those same cells so that the effects of stimulation could be unambiguously attributed to the identified cells. It was therefore important that the spontaneous and stimulation-evoked signals were very similar in shape and timing, showing that stimulation was effective and that it evoked the intended cell and not another adjacent cell. Figure 1C shows stimulus-evoked signals, and can be directly compared with spontaneous signals from the same cell and same electrode (Figure 1B). In all cases, and for all cells examined (*N* = 8), the spontaneous and evoked spikes on the same electrode looked the same (had the same shape), meaning that the intended cell was triggered. The timing was also the same, giving further evidence that the cell that had been targeted was the same one that was evoked. In Figure 1C, the additional shape at the very beginning of the spike is the last part of the stimulation artifact. Since the stimulating and recording electrodes were fairly close to each other, the stimulation artifact ended just before the evoked signal began. In general, the stimulation artifact could be large, especially on electrodes close to the stimulation point and in the channels. It was identified based on both timing and shape and had a bipolar (positive then negative) double square wave shape. If the evoked response did not overlap in time with the artifact, which was generally the case, the artifact could simply be ignored (analysis of the signal started after the artifact). In the rare cases, where there was an overlap, an algorithm using local curve fitting (Wagenaar and Potter, 2002) was used to extract the beginning of the signal from the underlying artifact.

### Response to stimulation was qualitatively similar for all cells

**Figure 2:**
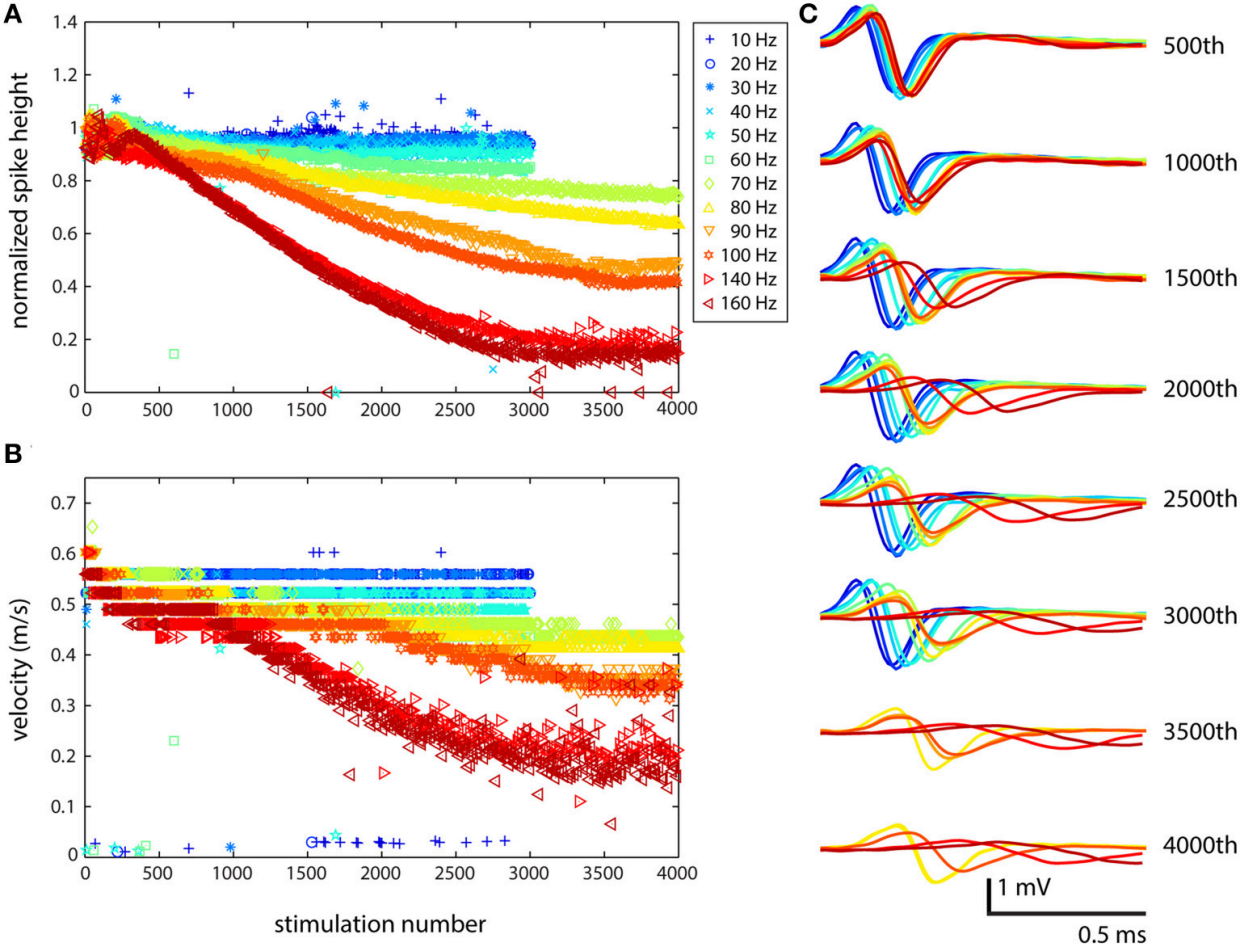
**Stimulation at medium to high frequencies results in highly altered axonal waveforms**. The cell was stimulated from 10 to 160 Hz, and because of the large waveforms in the channels, it was possible to very clearly observe the changes in waveform. **(A)** Large effects to the spike shape resulting from stimulation were not observed until stimulation at 80 Hz (yellow). At higher frequencies and following a larger number of stimulations, the effects were more dramatic. **(B)** Propagation velocity was also affected by stimulation: propagation slowed down at higher frequencies. **(C)** The change in shape of the waveform from top (500th stimulation) to bottom (4000th stimulation) shows the stereotypical behavior: decrease in spike height, increase in spike width, and increase in latency. The electrode from which these data were recorded is designated in Figure 1B with a black asterisk. Similar results were obtained for all electrodes in channels that were recorded from. Color corresponds to stimulation frequency.

The response of cells to stimulation was qualitatively the same: with increasing stimulation frequency and number of stimuli, the spike shape changed and the latency increased. Figure 2 shows the results of one set of stimulations based on the cell identified in Figure 1. Although, the exact behavior of each cell stimulated showed some variability, the results shown in Figure 2 demonstrate the usual characteristics seen for all stimulations that have been performed (*N* = 8 for the present study and *N* = 3 for pilot experiments performed previously). At lower frequencies, the axonal response was as expected: every stimulation resulted in a spike whose timing and shape was basically the same, except for slight differences due to jitter. At higher frequencies, several changes were observed: the spike height decreased, the spike width increased, and the latency increased as propagation velocity decreased. These changes were smooth and gradual rather than abrupt. This is shown explicitly in the waveforms in Figure 2C, while more generalized results are shown in Figures 2A,B. Such changes were observed across all electrodes that recorded from axons.

The increase in latency with successive stimulation could have been the result of delayed onset of spike initiation, so propagation velocity in the channels along several electrodes was also examined. Figure 2B shows one such velocity plot, which demonstrates that the change in latency was a real effect of the action potential slowing down, and not just delayed onset. To determine propagation velocity, the distance between the first electrode in the channel and the electrode whose spike shape is shown in Figure 2C (middle of the channel, shown in red in column 2 of Figure 1A and marked with a black asterisk) was divided by the difference in spike times at those electrodes, and this value was plotted. The assumption was that the axon grew straight through the channel and did not bend, turn back, or zig-zag, which is valid based on the spontaneous propagation velocity profile. At all stimulation frequencies, the velocity started at around 0.6 m/s, and then progressively decreased following more extensive stimulation. At lower frequencies, changes in velocity were small, resulting in a decrease to 0.5 m/s after several thousand stimulations. At higher frequencies, the effects on both propagation velocity and spike height were more drastic. At 70 Hz, the spike height was reduced to <80% of the original height following 4000 stimulations, while the velocity dropped to 0.4 m/s. At 140 and 160 Hz, the spike height decreased to <20% of the original height and the velocity fell to 0.2 m/s, only one third of the original velocity. Similar results were obtained for other cells. In a few cases, velocity increased at the beginning, and then went back down to its initial value or decreased below its initial value, depending on the stimulation frequency.

### Quantitative differences between cell responses were observed

**Figure 3:**
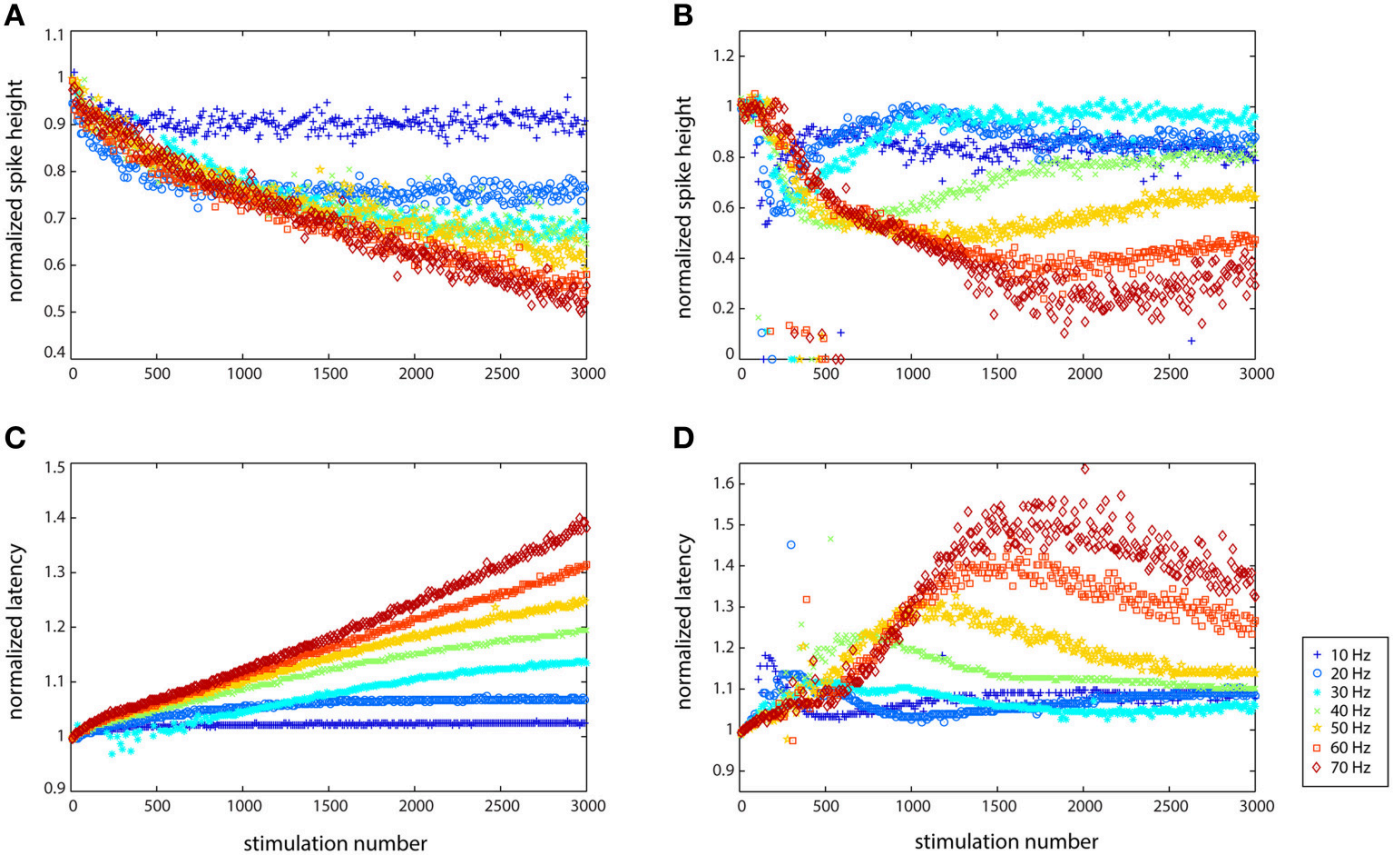
**Cells reacted differently to stimulation depending on their spontaneous characteristics (rate and spiking type) with regard to spike height (A,B) and spike latency (C,D)**. Data from two different cells is shown here. **(A,C)** Largely linear responses of the axon of one cell (cell 9123 in Figure 4), especially with regard to latency. **(B,D)** Adaptive behavior of another cell (cell 1237 in Figure 4), which responded non-linearly at every stimulation frequency that it was subjected to, but then recovered more (10 Hz) or less (70 Hz) in terms of both spike size and latency increase.

While the general trend illustrated in Figure 2 held for all of the cells examined (*N* = 11), there were both qualitative and quantitative differences in the responses of individual cells. For example, some cells responded in a non-monotonic fashion to stimulation. Figure 3 shows the results of stimulating two different cells from 10 to 70 Hz, showing the change in spike height and spike latency. While the axons of both cells were able to propagate the spikes at all seven frequencies, they modulated the spikes very differently. In Figure 3A, the spike height dips after several hundred stimulations and then continues to decrease linearly. The latency, Figure 3C, is linear, with each frequency showing a different rate of latency change: the slope of each line is different, where the smallest slope corresponds to the lowest frequency and the largest slope corresponds to the highest frequency. In Figure 3B very different behavior can be observed. At all frequencies, the first 100 spikes show no large changes, and then there is a large decrease in spike height, dropping below 60% of the original height, followed by a recovery, the extent of which depends on frequency, and which is mirrored by the change in latency. Latency (shown in Figure 3D) initially increased substantially and then decreased slowly. The only frequency at which full recovery was made is 30 Hz. As Figure 3B clearly shows, the onset of the original decrease happened after fewer stimulations at lower frequencies, but after a larger number of stimulations at higher frequencies. In fact, when the results are visualized by plotting spike height vs. time rather than stimulation number, all of the drops in the curves fall on top of one another: the cell reacts the same way after a given amount of time, about 10 s (100 stimulations at 10 Hz, 200 stimulations at 20 Hz, etc.), rather than after a given number of stimulation pulses. The data is plotted in this manner in Supplementary Figure 2 to better explain the phenomenon. These two cells show the two extremes in adaptive behavior that were observed. Other cells showed behavior that was somewhere in between a linear response and significant adaptation: some adaptation to stimulation followed by some recovery, depending on the frequency of stimulation.

**Figure 4:**
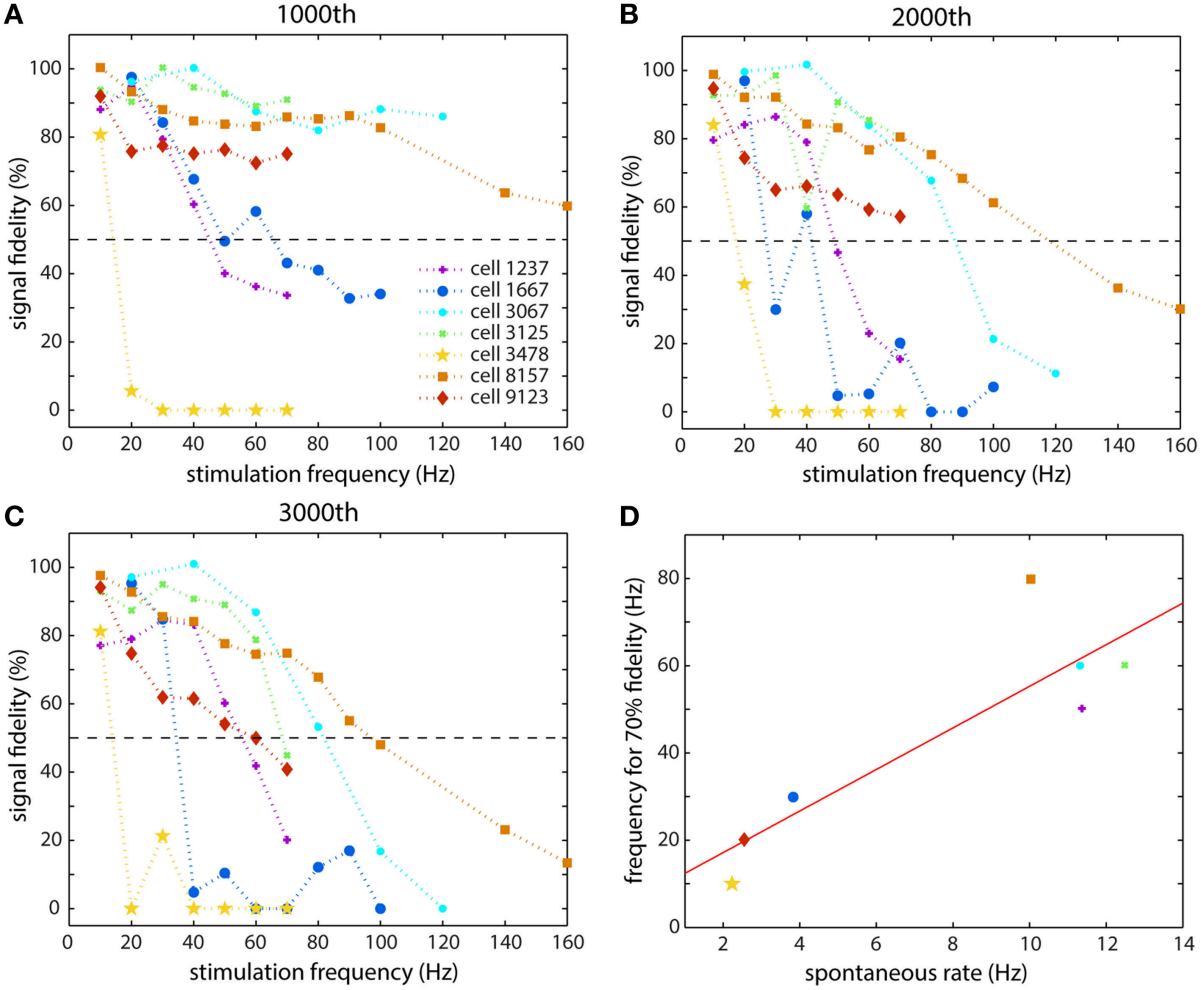
**Summary of the responses of seven different cells that were studied**. The spike fidelity (normalized spike height minus normalized latency) is shown for each cell at each stimulation frequency at different time points: **(A)** after 1000 stimulations, **(B)** after 2000 stimulations, and **(C)** after 3000 stimulations. **(D)** The spontaneous firing rate of a cell appeared to be related to the stimulation frequency that it could propagate faithfully. The abscissa is the average spontaneous spiking frequency of the cell and the ordinate is the frequency that the cell could propagate with 70% fidelity, taken from **(C)**.

The general results of all of the stimulations are shown in Figure 4, and they illustrate the diversity of responses from the different cells. Figures 4A–C show the relative fidelity with which an axon of each cell could propagate a spike at a given time point (following 1000, 2000, and 3000 stimulations, respectively). We define signal fidelity as the normalized spike height minus the normalized latency such that no change in either gives 100% fidelity. Because spike width was difficult to measure accurately, the signal fidelity was taken as a combination of the spike height and latency. As Figure 4 clearly demonstrates, different cells reacted differently to stimulation at the same frequency, and their responses also changed depending on the number of stimulations that the cell had received. The response of some cells (cell 3478, in yellow and cell 1667, in blue) was a sudden and rapid fall at a certain stimulation frequency, while the response of others was fairly linear (cell 8157, in orange). Some cells followed each other quite closely with increasing stimulation number (cells 3067, 3125, 8157) and others changed dramatically after 2000 and 3000 stimulations: cell 9123 follows the other three cells after 1000 stimulations, but then dropped off dramatically. The two cells shown in Figure 3 are cell 9123 (Figures 3A,C) and cell 1237 (Figures 3B,D). The recovery observed in Figures 3B,D can be seen in the steep slope in Figure 4A for cell 1237, which then becomes horizontal at lower frequencies in Figure 4C. Figure 4D summarizes the end effects (after 3000 stimulations) for each cell and will be discussed in the Discussion Section. The cell numbers refer to the electrodes that were used to stimulate the cell. Data from multiple electrodes per channel (ranging from 10 to 25 electrodes per channel), and multiple channels (depending on the number of axonal branches), were recorded and then examined for consistency and redundancy. One electrode, which was always located in a channel, and at least 50 μm from the channel entrance, and recording from beneath an axon, was then chosen as representative of the response of the cell being stimulated. Generally this electrode had a large amplitude spike to better enable spike shape analysis.

### Changes in [K^+^]_0_ affected axonal response

**Figure 5:**
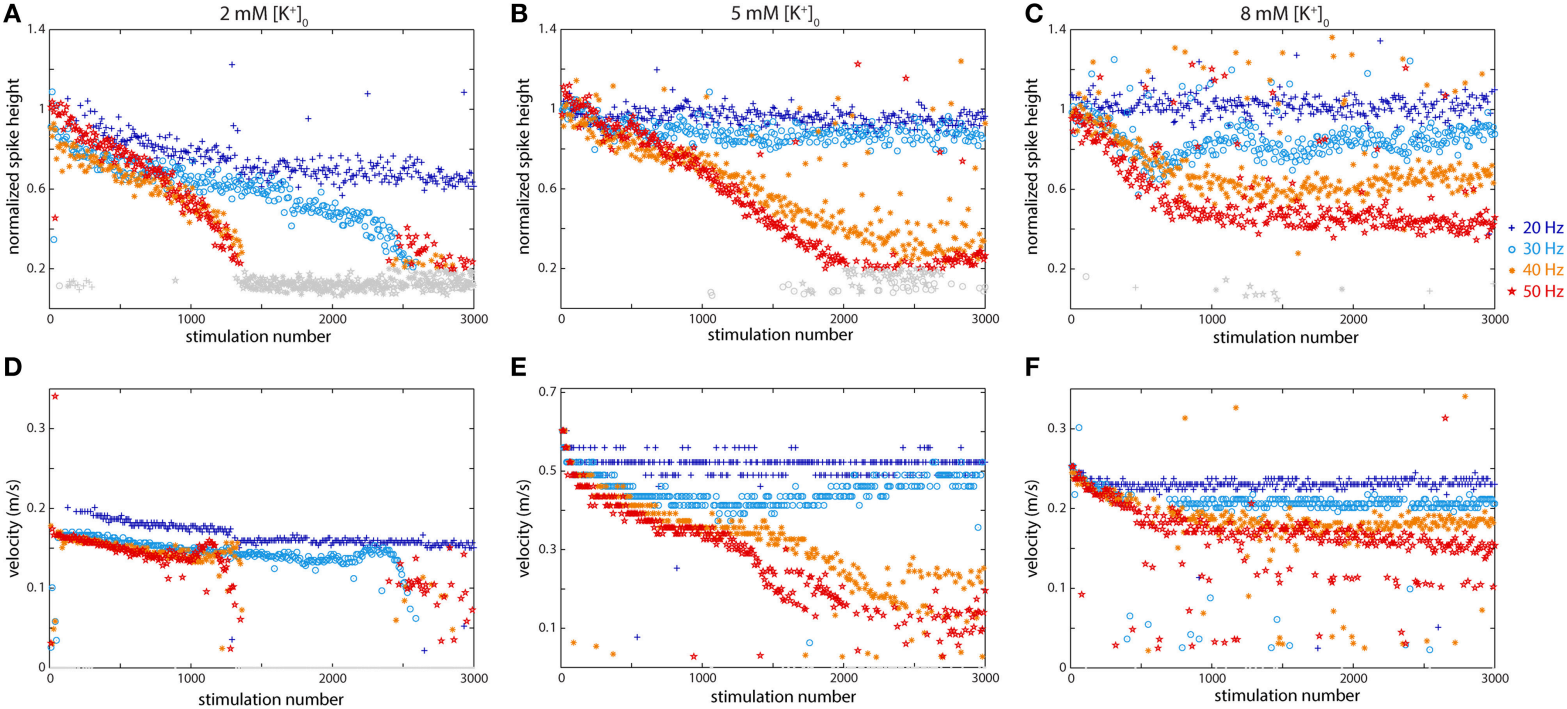
**Potassium concentration affects axonal propagation at higher frequencies including both the spike height (A–C) and the propagation velocity (D–F). (A,D)** Low K^+^ concentration 2 mM) caused much more dramatic effects and propagation failures than regular conditions. **(B,E)** Regular (5 mM) K^+^ concentration in the medium. **(C,F)** High K^+^ concentration (8 mM) also altered axonal behavior, but not as dramatically. Propagation velocity was affected by K^+^ concentration: both higher and lower [K^+^] slowed propagation.

Following the findings of several authors in both invertebrates and mammals (Grossman et al., 1979b; Poolos et al., 1987), we decided to change the initial concentration of potassium, [K^+^]_0_, in the extracellular medium in order to observe its effect on axonal propagation of spikes. These earlier experiments indicated that K+ build-up, resulting from stimulation, made an important contribution to the observed changes in propagation. The previous experiments were all performed using the standard aCSF medium, with a potassium concentration ([K^+^]_0_) of 5 mM. The plots in Figure 5 show data from two experiments where [K^+^]_0_ was changed: the left column (Figure 5A) shows the results of stimulation following a bath application of low [K^+^]_0_ medium (2 mM), while the right column (Figure 5C) shows the results of stimulation following a bath application of high [K^+^]_0_ medium (8 mM). The middle column (Figure 5B) shows results using the standard aCSF.

As Figure 5 shows, altering the potassium concentration had a dramatic effect on axonal propagation. Compared to the normal situation, the axon was not even able to faithfully propagate spikes at 20 Hz (spike height dropped to 60%, velocity greatly reduced), with lowered K^+^ levels, while at higher frequencies the conduction was even more severely affected. At all higher frequencies, from 30 to 50 Hz, there was drastic decline in spike size and a large decrease in velocity following 2500 (at 30 Hz) or 1000 (>30 Hz) stimulations. In all cases, the initial changes in spike height were linear, but then dropped off suddenly. With elevated K^+^ levels, the effects did not follow as clear of a trend, but again behavior was altered compared to standard conditions. Under normal conditions, both 20 and 30 Hz spikes propagated rather faithfully, but in high K^+^ medium, only 20 Hz spikes propagated faithfully while the 30 Hz spike height pattern resembles a sinusoid. On the other hand, higher frequency spikes propagated more faithfully in high K^+^ conditions than under normal conditions. Spike heights resulting from stimulation at 40 Hz and 60 Hz all showed a clear linear decrease in the normal medium, while in high K^+^, they all declined somewhat, but then plateaued around 50% (40 Hz) or 30% (50 Hz) of the original height. Changes in velocity were also observed in higher and lower K^+^ medium: in both cases the initial velocity was less than half of that in regular medium (Figure 5E). In low [K^+^]_0_, the velocity dramatically decreased at all stimulation frequencies except 20 Hz. In high [K^+^]_0_, the velocity profiles were much more uniform and did not change much at the different frequencies. In Figure 5, spike heights that fell to below 20% of the original height are shown in gray and velocities were not calculated for these since they were difficult to differentiate from the noise.

### Differential modulation was observed along axonal branches

**Figure 6:**
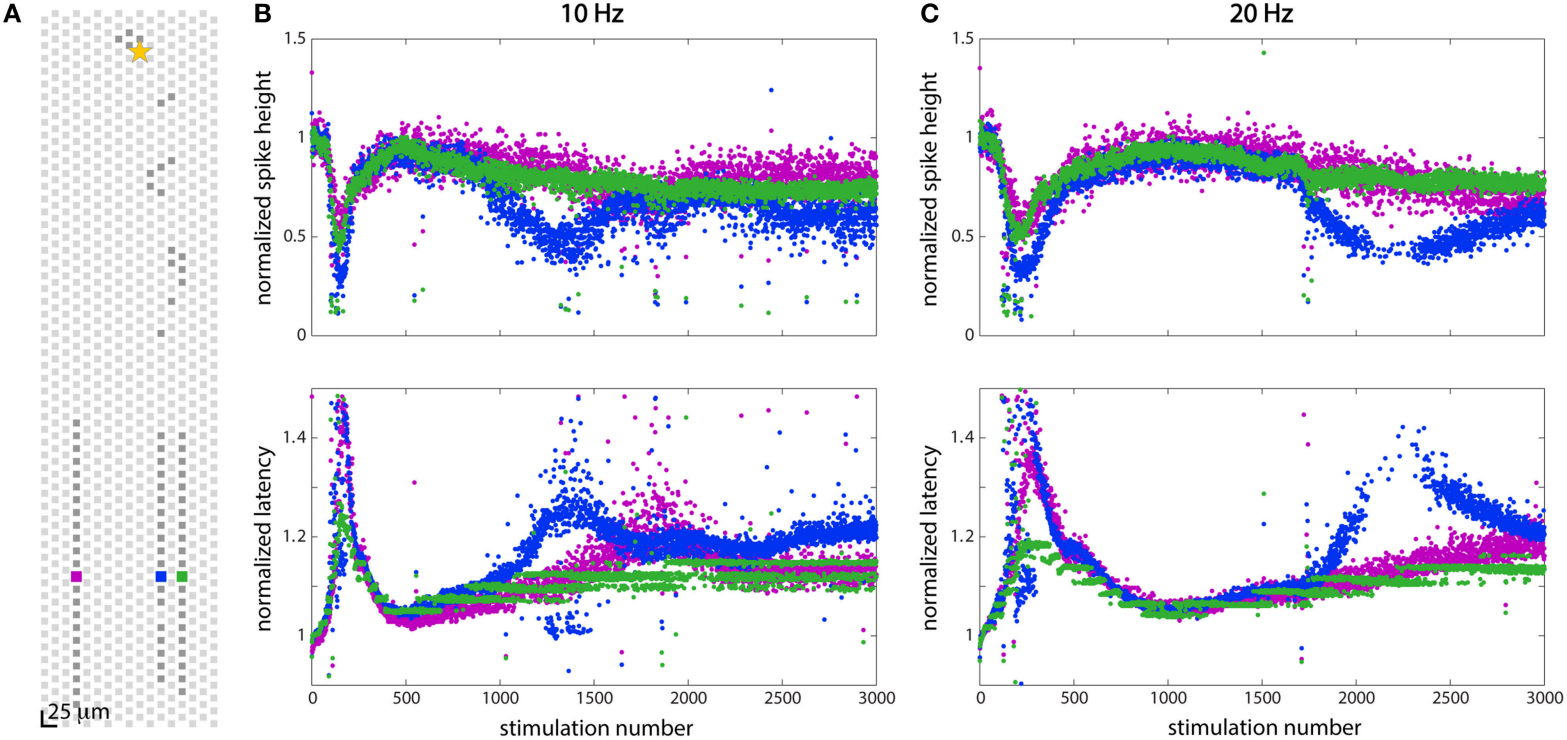
**Stimulation at 10 Hz and 20 Hz showed different effects in two axonal branches. (A)** Position of electrodes in three channels from which spikes were recorded. **(B)** Responses to stimulation at 10 Hz at the three electrodes shown in **(A)**. **(C)** Responses to stimulation at 20 Hz at the three electrodes shown in **(A)**. At both 10 and 20 Hz, all three axonal branches showed slightly different responses to stimulation, although the blue branch showed the most dramatic effects. All three branches display the same adaptation characteristics following 150 (at 10 Hz) or 200 (at 20 Hz) stimulations, but additional changes in the wave forms are seen in some branches while not in others.

While the response to stimulation was often fairly uniform across a given cell, differential effects along different axonal branches belonging to the same cell were observed as well. Figure 6 shows a differential response in three axonal branches to the same stimulus at two different stimulation frequencies. Figure 6A shows the electrode layout: three electrodes (one in each channel) were chosen and they are shown in purple (in the left channel), blue (in the middle channel), and green (in the right channel). Figures 6B,C shows data recorded from the chosen electrodes when the soma (depicted by a star in Figure 6A) was stimulated at 10 Hz (20 Hz). This is the same neuron that was shown in Figures 3B,D. All of the branches showed the same adaptive behavior that is depicted in Figures 3B,D, but the effects were more pronounced in some branches than in others. While the purple and green signals generally track each other quite closely, the signals recorded from the blue branch show different behavior. Closer inspection of the purple and green branches in Figure 6B reveals that these are not identical either, but that the purple branch shows some troughs (in spike height) and peaks (in latency), similar to the blue branch.

## Discussion

The major goal of this study was to observe the effect of high frequency stimulation on axonal propagation: to recognize conserved or average effects along many different axons and to look for differences between cells as well as differential effects that could only be seen, e.g., along one axonal branch but not another belonging to the same neuron. The effects illustrated in Figure 2 through Figure 6, namely the decreasing height, broadening width, and increasing latency were observed for all of the neurons that were stimulated, and this behavior is well documented in many different preparations ranging from hippocampus to sensory fibers (Poolos et al., 1987; Thalhammer et al., 1994; Weidner et al., 2003; De Col et al., 2008). Observations of spike shape changes have been made by many different researchers as a consequence of high frequency stimulation, as has been described, as well as somatic depolarization (Kole et al., 2007), and other mechanisms, and there is conflicting evidence as to the consequences. Data from granule cells indicate that spike broadening leads to increases in post-synaptic Ca^2+^ influx, which in turn increases synaptic strength (Sabatini and Regehr, 1997). At hippocampal mossy fiber synapses, on the other hand, decreases in action potential height reduce presynaptic Ca^2+^ inflow, and the total charge (width × height) is most important in determining the Ca^2+^ current (Bischofberger et al., 2002).

Eight cells on four different chips were examined, while the same effects were observed for three other cells on two different chips during earlier exploratory experiments (data not shown). One issue with detecting spikes whose amplitudes decreased over time was that, at some point, the signal could no longer be extracted from the noise, but may still have been present. The larger the initial signal height, such as in Figure 2, the easier it was to detect, even when it fell to 10–20% of its original size. When the various parameters were normalized by dividing by their spontaneous spike heights, however, it was possible to compare signals of different size in a useful way, by noting the time when the spike height fell to, e.g., 50% of its original size or the latency doubled. Therefore, even though the original size of the detected spike depends on many factors, including the proximity and sealing of the axon to the electrode, the amount of other tissue in the channel, the position of the electrode in the channel, etc., the influence of some of these factors could be reduced in the analysis by using normalized parameters.

In spite of the many advantages created by the microelectrode array and the channel device, a number of potential pitfalls also exist, and they need to be carefully assessed when evaluating the data. Extracellular stimulation, especially at high frequencies, can lead to stimulation failures, which could be interpreted as propagation failures. Stimulation failures also mean that the intended stimulation frequency will not be achieved consistently during an experiment, and this needs to be taken into account. When studying cells with only one visible axonal segment, we assumed that failures along the axon were the result of stimulation failure rather than propagation failure. Figure 5A, for example, shows some stimulation failures in gray (between 1 and 500 stimulations), where the waveform could not be detected. Likewise, if more than one axonal branch displayed failures, we assumed these were stimulation failures, since this is a much more likely scenario than selective propagation failure. As a result of the fact that several axons could grow through the same channel, along with our use of extracellular electrodes, we again observed addition of wave forms in channels, as in our previous work (Lewandowska et al., 2015). Such additions could, without careful analysis, be falsely interpreted as real changes in the propagating spike. Due to the large number of available electrodes and wealth of recordings, it was possible to detect such linearly added spike forms so that we were able to detect cases of signal superposition and could rule out the presence of a more complex phenomenon. All these complications meant that data analysis had to be performed thoughtfully, but as long as this was done, the advantages of the large spikes and multiplicity of electrodes strongly enabled data analysis.

Combining the results of stimulation of many different cells, it appears that the axons of different cells reacted in a similar qualitative way but in different quantitative ways to a given stimulation frequency. The representative responses of the cells with respect to signal fidelity, as seen in Figure 4, appear very different. Although, not in the central nervous system, a similar type of behavior is known in sensory nerves where activity induced velocity changes are used to classify nerves into different types (Weidner et al., 1999, 2003). For example, nociceptor and cold fibers exhibit very different latency increases as a result of stimulation (Thalhammer et al., 1994).

One hypothesis for why different neurons reacted differently at various frequencies may include that there is some relationship between the spontaneous spiking frequency and spiking behavior, e.g., tonic spiking or bursting, and the spiking frequency that the neuron is able to propagate. The spontaneous behavior would be the result of the distribution of ion channels along the cell and its axonal arbor. (Bean, 2007) In our data, it appears that cells whose spontaneous spike rate was higher were able to propagate higher frequency signals along their axons, while cells that fired less often were more affected by high frequency stimulation and failed to propagate faithfully at higher frequencies. We looked at what frequencies of stimulation an axon could faithfully propagate for the full duration of stimulation, 3000 stimulations. Faithful propagation was defined as a combined spike height and latency change of no more than 70% of the original value, as shown in Figure 4C. Of the eight cells that we studied in depth, three had spontaneous spiking rates below 4 Hz, and these three cells were also the ones least able to propagate higher frequency spikes (up to 30 Hz). The two most inactive cells had spontaneous rates of 2.2 and 2.5 Hz, and these cells were only able to faithfully propagate 10 and 20 Hz spikes. On the other hand, the most active cell had a spontaneous rate of 12.5 Hz, and was able to faithfully propagate 10 to 70 Hz spikes. These results are summarized in Figure 4D. It is quite probable that these cells came from two (or more) different populations (Brodmann, 1909), since there is a marked separation between them in Figure 4D. Since we did not perform any micro-dissection of the cortex, several cortical regions were represented in our cell population. Examining their instantaneous spiking rates indicated that the cells on the right had a slightly smaller average inter-spike interval (ISI) than the cells on the left (6−7 × 10^−2^ s vs. 1 × 10^−1^ s), but the major difference between the groups was the spontaneous rate, the total number of spikes divided by the total time that the spontaneous activity was recorded, was significantly higher for the cells that were able to propagate higher frequencies more faithfully. The cell in Figures 3B,D, that showed the adaptive behavior, was also an interesting case, since it only showed bursting behavior, while all of the other cells showed regular spiking behavior. At a rate of about 1 Hz, it would burst 8–20 times at a very high frequency, often exceeding 100 Hz, and then would be silent before bursting again. It is possible that the large dip in spike height that was observed after 100 stimulations is a manifestation of a near-failure mechanism since the cell spontaneously never spiked that many times consecutively. As far as we know, this type of analysis has not been reported for CNS neurons, although differences in action potential shapes between cells in cortical layer 2/3 and cortical layer 5 have been observed (de Polavieja et al., 2005).

It was reported by several authors (Grossman et al., 1979a; Poolos et al., 1987) that levels of extracellular potassium were elevated following stimulation at medium to high frequencies, and that changing the concentration of extracellular potassium could exacerbate (at higher concentrations) or alleviate (at lower concentrations) the effects of stimulation by causing a small depolarization or hyperpolarization, respectively, of the cell. They observed that although elevation of potassium concentration did not exactly recreate the effects of high frequency stimulation, it did mimic some of the observed behavior. Our experiments with changing extracellular potassium levels from the usual 5 mM down to 2 mM and up to 8 mM are shown in Figure 5, and they are somewhat inconsistent with what was previously reported by some (Grossman et al., 1979b; Poolos et al., 1987), but very consistent with observations made by others (Meeks et al., 2005; De Col et al., 2008), although direct comparison is difficult because of the different cell types and media used. Poolos et al. observed a somewhat flat response between concentrations ranging from 3 to 6 mM, but a significant increase in latency and decrease in spike height at 8 mM concentrations when stimulating at 0.17 Hz (Poolos et al., 1987). Grossman et al. only looked at increased K^+^ concentrations from a level that was initially higher than what we started with, and they saw a clear decrease in spike height when stimulating at 30 Hz, which lead to conduction block (Grossman et al., 1979b). De Col et al. focused primarily on latency, which increased substantially at higher K^+^ concentrations and decreased at lower K^+^ concentrations (De Col et al., 2008). Our experiments with both lower and higher potassium concentrations were repeated for three other cells, and in all cases the results were very similar: lowering extracellular potassium drastically exacerbated the effects of stimulation, so much so that the neuron in Figure 5 could only propagate signals at 20 Hz, and all higher frequency pulses propagated very poorly.

Evidence of differences in propagation between axonal branches has been demonstrated using large invertebrate axons as their model systems (Grossman et al., 1979a). Experiments showed that geometrical differences in axonal branches could generally explain propagation changes and propagation failures, with thicker branches failing before thinner branches because the load of the thicker branch was too large to become depolarized (Parnas and Segev, 1979; Grossman et al., 1979a,b). Similarly, antidromic stimulation has been shown to fail to activate the somatic compartment of a neuron for the same reason. Although, we do not have microscopy data on the cell shown in Figure 6, we know that the propagation velocity along the blue branch was slower than along the purple and green branches suggesting that the blue branch could be thinner than the other branches (Goldstein and Rall, 1974; Waxman and Swadlow, 1977), although conduction velocity has been known to vary for other reasons, such as conduction history (Swadlow and Waxman, 1975), as we have seen. Since a thinner axon will have a larger surface-to-volume ratio, effects of membrane channels will be more apparent in thinner axons. Since we also saw the opposite effects of [K^+^] on axonal propagation: rather than increasing the possibility of failure at lower frequency, higher potassium concentrations appeared to be protective to the axon, while lowered concentrations produced more failures. Following the logic of Grossman et al. (1979b), and assuming, for example, a Na^+^-dependent K^+^ current (Bader et al., 1985), repetitive stimulation should cause a buildup of Na^+^ in the thinner branch before the thicker branch, thus activating the K^+^ current there first, and reducing the excitability of the thinner branch, as we have observed.

Certainly the interpretation of all these results depends very much on the cell type, which is related to the distribution of various ion channels, among other things. Grossman et al. argued that the origin of the observed effects (in their case clear propagation failure preferentially along one branch of the nerve innervating deep abdominal extensor muscles of the spiny lobster) lay in a failure of the Na^+^/K^+^-ATPase to effectively clear excess K^+^ from the peri-axonal space, as they were able to mimic effects of high frequency stimulation by both increasing extracellular [K^+^] and blocking the Na^+^/K^+^-ATPase with ouabain (Grossman et al., 1979a). Morita et al. found that Na^+^/K^+^-ATPase hyperpolarization was responsible for changes resulting from post-tetanic stimulation of myelinated lizard axons, such as reductions in conduction velocity and reduced safety factor (Morita et al., 1993). De Col et al. contradict these findings, and argue that their observations (changes in latency following repetitive stimulation in the cranial meninges) can be attributed to Na^+^ channel inactivation (De Col et al., 2008). De Polavieja et al. state that activity-dependent reductions in spike amplitude and spike broadening are the result of sodium and potassium channels, but they make no further qualifications as to which ones are most important (de Polavieja et al., 2005). There is also evidence that Ca^2+^ channels, which act to hyperpolarize the cell, are involved in shaping the action potential, especially during repetitive stimulation (Pineda et al., 1998; Stewart and Foehring, 2001).

## Conclusion

The utility and advantages of our axonal isolation device coupled with the HD-MEA have been demonstrated in the context of axonal information processing at various medium and high stimulation frequencies. The amplification effect of the channel structures enabled pairing a neuronal soma with its processes, often including several axonal branches that had grown into different channels, by using spontaneous spiking activity. Stimulation near the soma evoked the same cell that was observed using spontaneous spiking, and showed that those cells reacted to stimulation in a reliable way. Moreover, the signal characteristics of spontaneous and stimulated activity were found to be very similar. Medium to high frequency stimulation was performed on the cells, and the responses to stimulation along the axons were studied. All of the cells showed the same basic response to stimulation: following a larger number of stimuli and at higher frequencies, spike heights decreased, spike widths increased, and latencies increased as propagation velocities decreased. In a couple of cases, differences in modulation between axonal branches were observed. In general, the cells showed similar qualitative effects to one another, but were able to propagate spikes at very different frequencies. Some cells were unable to propagate spikes already at 30 Hz, while others were able to propagate spikes at 140 Hz, although with drastically altered shape and timing. The reason for these differences appears to correlate with the spontaneous spiking behavior of each cell, where cells with higher spontaneous spiking rates were able to maintain propagation at higher frequencies, while cells with lower spontaneous rates showed propagation failure.

High frequency bursting is a known phenomenon *in vitro* and *in vivo*. The changes in spike shape and timing that we observed followed a clear trend, which was that the higher the stimulation frequency, the sooner (in time) the onset of observed effects. Therefore, at very high frequencies, such as have been observed in both dissociated cells and slice preparations, only several to tens of spikes could be enough to bring about the effects observed: Rather than observing a certain signal distortion after 100 spikes at, for example, 70 Hz, signal distortion may be observed after only 10 spikes at 100 or 150 Hz, a scenario that might be realistic during a burst. Shimba et al. have recently reported changes in conduction velocity during spontaneous bursts using channel devices *in vitro* (Shimba et al., 2015). Likewise, a number of different authors have used natural stimulation sequences to evoke behavior to show that the observed changes can happen under normal circumstances (Grossman et al., 1979a). The next important questions to ask are: what are the consequences of the observed behavior? Do these mechanisms really influence information processing in the brain? Based on the literature we have discussed, it seems that it may be so. Clearly, these interesting results require additional studies, some of which will certainly be enabled by the developments in microfabrication, such as those described in the present work.

## Author contributions

ML designed and carried out experiments, wrote software, analyzed data, and wrote the manuscript. MR helped with experiment concept and design, contributed software, and contributed to the manuscript. DJ contributed with software and data analysis, and contributed to the manuscript. JM helped with hardware and software, and contributed to the manuscript. AH helped with data interpretation, and contributed to the manuscript.

### Conflict of interest statement

The authors declare that the research was conducted in the absence of any commercial or financial relationships that could be construed as a potential conflict of interest. The reviewer HS declared a shared affiliation, though no other collaboration, with one of the authors ML to the handling Editor, who ensured that the process nevertheless met the standards of a fair and objective review.

## References

[B1] Bader C. R. Bernheim L. Bertrand D. (1985). Sodium-activated potassium current in cultured avian neurones. Nature 317, 540–542. 10.1038/317540a0 2413369

[B2] Bakkum D. J. Frey U. Radivojevic M. Russell T. Müller J. Fiscella M.. (2013). Tracking axonal action potential propagation on a high-density microelectrode array across hundreds of sites. Nat. Commun. 4:2181. 10.1038/ncomms3181 23867868 PMC5419423

[B3] Bean B. P. (2007). The action potential in mammalian central neurons. Nat. Rev. Neurosci. 8, 451–465. 10.1038/nrn2148 17514198

[B4] Berdondini L. Imfeld K. Maccione A. Tedesco M. Neukom S. Koudelka-Hep M.. (2009). Active pixel sensor array for high spatio-temporal resolution electrophysiological recordings from single cell to large scale neuronal networks. Lab Chip 9, 2644–2651. 10.1039/b907394a 19704979

[B5] Bischofberger J. Engel D. Li L. Geiger J. R. Jonas P. (2006). Patch-clamp recording from mossy fiber terminals in hippocampal slices. Nat. Protoc. 1, 2075–2081. 10.1038/nprot.2006.312 17487197

[B6] Bischofberger J. Geiger J. R. Jonas P. (2002). Timing and efficacy of Ca2+ channel activation in hippocampal mossy fiber boutons. J. Neurosci. 22, 10593–10602. 12486151 10.1523/JNEUROSCI.22-24-10593.2002 PMC6758411

[B7] Brodmann K. (1909). Vergleichende Lokalisationslehre der Großhirnrinde. Leipzig: Barth.

[B8] Campenot R. B. (1977). Local control of neurite development by nerve growth factor. Proc. Natl. Acad. Sci. U.S.A. 74, 4516–4519. 10.1073/pnas.74.10.4516 270699 PMC431975

[B9] Campenot R. B. (2009). NGF uptake and retrograde signaling mechanisms in sympathetic neurons in compartmented cultures. Cell Biol. Axon 4, 141–158. 10.1007/400_2009_7 19343309

[B10] Coombs J. S. Curtis D. R. Eccles J. C. (1957). The interpretation of spike potentials of motoneurones. J. Physiol. 139, 198–231. 10.1113/jphysiol.1957.sp005887 13492209 PMC1358725

[B11] De Col R. Messlinger K. Carr R. W. (2008). Conduction velocity is regulated by sodium channel inactivation in unmyelinated axons innervating the rat cranial meninges. J. Physiol. 586, 1089–1103. 10.1113/jphysiol.2007.145383 18096592 PMC2375633

[B12] de Polavieja G. G. Harsch A. Kleppe I. Robinson H. P. Juusola M. (2005). Stimulus history reliably shapes action potential waveforms of cortical neurons. J. Neurosci. 25, 5657–5665. 10.1523/JNEUROSCI.0242-05.2005 15944394 PMC6724966

[B13] Debanne D. Boudkkazi S. (2010). New insights in information processing in the axon, in New Aspects of Axonal Structure and Function, eds Feldmeyer D. Lübke J. H. R. (New York, NY; Dordrecht; Heidelberg; London: Springer) 55–83.

[B14] Debanne D. (2004). Information processing in the axon. Nat. Rev. Neurosci. 5, 304–316. 10.1038/nrn1397 15034555

[B15] Dworak B. J. Wheeler B. C. (2009). Novel MEA platform with PDMS microtunnels enables the detection of action potential propagation from isolated axons in culture. Lab Chip 9, 404–410. 10.1039/B806689B 19156289 PMC2790813

[B16] Engel D. Jonas P. (2005). Presynaptic action potential amplification by voltage-gated Na+ channels in hippocampal mossy fiber boutons. Neuron 45, 405–417. 10.1016/j.neuron.2004.12.048 15694327

[B17] Fatt P. (1957). Sequence of events in synaptic activation of a motoneurone. J. Neurophysiol. 20, 61–80. 13398851 10.1152/jn.1957.20.1.61

[B18] Fawcett J. W. (1992). Intrinsic neuronal determinants of regeneration. Trends Neurosci. 15, 5–8. 10.1016/0166-2236(92)90338-9 1374956

[B19] Fiscella M. Franke F. Farrow K. Müller J. Roska B. da Silveira R.. (2015). Visual coding with a population of direction-selective neurons. J. Neurophysiol. 114, 2485–2499. 10.1152/jn.00919.2014 26289471 PMC4620130

[B20] FitzGerald J. J. Lacour S. P. McMahon S. B. Fawcett J. W. (2008). Microchannels as axonal amplifiers. IEEE Trans. Biomed. Eng. 55, 1136–1146. 10.1109/TBME.2007.909533 18334406

[B21] Frey U. Egert U. Heer F. Hafizović S. Hierlemann A. (2009). Microelectronic system for high-resolution mapping of extracellular electric fields applied to brain slices. Biosens. Bioelectron. 24, 2191–2198. 10.1016/j.bios.2008.11.028 19157842

[B22] Frey U. Šedivý J. Heer F. Pedron R. Ballini M. Müller J. (2010). Switch-matrix-based high-density microelectrode array in CMOS technology. IEEE J. Solid State Circuits 45, 467–482. 10.1109/JSSC.2009.2035196

[B23] Fuortes M. Frank K. Becker M. C. (1957). Steps in the production of motoneuron spikes. J. Gen. Physiol. 40, 735–752. 10.1085/jgp.40.5.735 13428986 PMC2147645

[B24] Geiger J. R. Jonas P. (2000). Dynamic control of presynaptic Ca 2+ inflow by fast-inactivating K+ channels in hippocampal mossy fiber boutons. Neuron 28, 927–939. 10.1016/S0896-6273(00)00164-1 11163277

[B25] Goldstein S. S. Rall W. (1974). Changes of action potential shape and velocity for changing core conductor geometry. Biophys. J. 14, 731–757. 10.1016/S0006-3495(74)85947-3 4420585 PMC1334570

[B26] Grossman Y. Parnas I. Spira M. (1979a). Differential conduction block in branches of a bifurcating axon. J. Physiol. 295, 283–305. 10.1113/jphysiol.1979.sp012969 521937 PMC1279046

[B27] Grossman Y. Parnas I. Spira M. (1979b). Mechanisms involved in differential conduction of potentials at high frequency in a branching axon. J. Physiol. 295, 307–322. 10.1113/jphysiol.1979.sp012970 521940 PMC1279047

[B28] Häusser M. Stuart G. Racca C. Sakmann B. (1995). Axonal initiation and active dendritic propagation of action potentials in substantia nigra neurons. Neuron 15, 637–647. 10.1016/0896-6273(95)90152-3 7546743

[B29] Hill D. N. Mehta S. B. Kleinfeld D. (2011). Quality metrics to accompany spike sorting of extracellular signals. J. Neurosci. 31, 8699–8705. 10.1523/JNEUROSCI.0971-11.2011 21677152 PMC3123734

[B30] Hodgkin A. L. Huxley A. F. (1952). A quantitative description of membrane current and its application to conduction and excitation in nerve. J. Physiol. 117, 500. 10.1113/jphysiol.1952.sp004764 12991237 PMC1392413

[B31] Huxley A. Stämpeli R. (1949). Evidence for saltatory conduction in peripheral myelinated nerve fibres. J. Physiol. 108, 315–339. 10.1113/jphysiol.1949.sp004335 18144923 PMC1392492

[B32] Imfeld K. Neukom S. Maccione A. Bornat Y. Martinoia S. P. Berdondini L.. (2008). Large-scale, high-resolution data acquisition system for extracellular recording of electrophysiological activity. IEEE Trans. Biomed. Eng. 55, 2064–2073. 10.1109/TBME.2008.919139 18632369

[B33] Ishikawa D. Takahashi N. Sasaki T. Usami A. Matsuki N. Ikegaya Y. (2010). Fluorescent pipettes for optically targeted patch-clamp recordings. Neural Netw. 23, 669–672. 10.1016/j.neunet.2010.02.004 20223634

[B34] Kole M. H. Letzkus J. J. Stuart G. J. (2007). Axon initial segment Kv1 channels control axonal action potential waveform and synaptic efficacy. Neuron 55, 633–647. 10.1016/j.neuron.2007.07.031 17698015

[B35] Kress G. J. Mennerick S. (2009). Action potential initiation and propagation: upstream influences on neurotransmission. Neuroscience 158, 211–222. 10.1016/j.neuroscience.2008.03.021 18472347 PMC2661755

[B36] Lambacher A. Vitzthum V. Zeitler R. Eickenscheidt M. Eversmann B. Thewes R. (2011). Identifying firing mammalian neurons in networks with high-resolution multi-transistor array (MTA). Appl. Phys. A 102, 1–11. 10.1007/s00339-010-6046-9

[B37] Lewandowska M. K. Bakkum D. J. Rompani S. B. Hierlemann A. (2015). Recording large extracellular spikes in microchannels along many axonal sites from individual neurons. PLoS ONE 10:e0118514. 10.1371/journal.pone.0118514 25734567 PMC4348166

[B38] Meeks J. P. Jiang X. Mennerick S. (2005). Action potential fidelity during normal and epileptiform activity in paired soma-axon recordings from rat hippocampus. J. Physiol. 566, 425–441. 10.1113/jphysiol.2005.089086 15890699 PMC1464751

[B39] Morita K. David G. Barrett J. N. Barrett E. F. (1993). Posttetanic hyperpolarization produced by electrogenic Na (+)-K+ pump in lizard axons impaled near their motor terminals. J. Neurophysiol. 70, 1874–1884. 8294960 10.1152/jn.1993.70.5.1874

[B40] Pan L. Alagapan S. Franca E. DeMarse T. Brewer G. J. Wheeler B. C.. (2013). Large extracellular spikes recordable from axons in microtunnels. IEEE Trans. Neural Syst. Rehabil. Eng. 22, 453–459. 10.1109/TNSRE.2013.2289911 24240004 PMC4013201

[B41] Parnas I. Segev I. (1979). A mathematical model for conduction of action potentials along bifurcating axons. J. Physiol. 295, 323–343. 10.1113/jphysiol.1979.sp012971 521942 PMC1279048

[B42] Pineda J. C. Waters R. S. Foehring R. C. (1998). Specificity in the interaction of HVA Ca2+ channel types with Ca2+-dependent AHPs and firing behavior in neocortical pyramidal neurons. J. Neurophysiol. 79, 2522–2534. 9582225 10.1152/jn.1998.79.5.2522

[B43] Poolos N. P. Mauk M. D. Kocsis J. (1987). Activity-evoked increases in extracellular potassium modulate presynaptic excitability in the CA1 region of the hippocampus. J. Neurophysiol. 58, 404–416. 3655875 10.1152/jn.1987.58.2.404

[B44] Qing Q. Pal S. K. Tian B. Duan X. Timko B. P. Lieber C. M.. (2010). Nanowire transistor arrays for mapping neural circuits in acute brain slices. Proc. Natl. Acad. Sci. U.S.A. 107, 1882–1887. 10.1073/pnas.0914737107 20133836 PMC2808222

[B45] Sabatini B. L. Regehr W. G. (1997). Control of neurotransmitter release by presynaptic waveform at the granule cell to Purkinje cell synapse. J. Neurosci. 17, 3425–3435. 9133368 10.1523/JNEUROSCI.17-10-03425.1997 PMC6573699

[B46] Sasaki T. Matsuki N. Ikegaya Y. (2011). Action-potential modulation during axonal conduction. Science 331, 599–601. 10.1126/science.1197598 21292979

[B47] Sasaki T. Matsuki N. Ikegaya Y. (2012a). Effects of axonal topology on the somatic modulation of synaptic outputs. J. Neurosci. 32, 2868–2876. 10.1523/JNEUROSCI.5365-11.2012 22357869 PMC6621900

[B48] Sasaki T. Matsuki N. Ikegaya Y. (2012b). Targeted axon-attached recording with fluorescent patch-clamp pipettes in brain slices. Nat. Protoc. 7, 1228–1234. 10.1038/nprot.2012.061 22653161

[B49] Shimba K. Sakai K. Isomura T. Kotani K. Jimbo Y. (2015). Axonal conduction slowing induced by spontaneous bursting activity in cortical neurons cultured in a microtunnel device. Integr. Biol. 7, 64–72. 10.1039/C4IB00223G 25418582

[B50] Shu Y. Hasenstaub A. Duque A. Yu Y. McCormick D. A. (2006). Modulation of intracortical synaptic potentials by presynaptic somatic membrane potential. Nature 441, 761–765. 10.1038/nature04720 16625207

[B51] Stewart A. E. Foehring R. C. (2001). Effects of spike parameters and neuromodulators on action potential waveform-induced calcium entry into pyramidal neurons. J. Neurophysiol. 85, 1412–1423. 11287465 10.1152/jn.2001.85.4.1412

[B52] Swadlow H. Waxman S. (1975). Observations on impulse conduction along central axons. Proc. Natl. Acad. Sci. U.S.A. 72, 5156–5159. 10.1073/pnas.72.12.5156 1061101 PMC388895

[B53] Taylor A. M. Blurton-Jones M. Rhee S. W. Cribbs D. H. Cotman C. W. Jeon N. L. (2005). A microfluidic culture platform for CNS axonal injury, regeneration and transport. Nat. Methods 2, 599–605. 10.1038/nmeth777 16094385 PMC1558906

[B54] Taylor A. M. Rhee S. W. Tu C. H. Cribbs D. H. Cotman C. W. Jeon N. L. (2003). Microfluidic multicompartment device for neuroscience research. Langmuir 19, 1551–1556. 10.1021/la026417v 20725530 PMC2923462

[B55] Thalhammer J. G. Raymond S. A. Popitz-Bergez F. A. Strichartz G. R. (1994). Modality-dependent modulation of conduction by impulse activity in functionally characterized single cutaneous afferents in the rat. Somatosens. Mot. Res. 11, 243–257. 10.3109/08990229409051392 7887056

[B56] Wagenaar D. A. Potter S. M. (2002). Real-time multi-channel stimulus artifact suppression by local curve fitting. J. Neurosci. Methods 120, 113–120. 10.1016/S0165-0270(02)00149-8 12385761

[B57] Waxman S. G. Swadlow H. A. (1977). The conduction properties of axons in central white matter. Progr. Neurobiol. 8, 297–324. 10.1016/0301-0082(77)90009-0 335441

[B58] Weidner C. Schmelz M. Schmidt R. Hansson B. Handwerker H. Torebjörk H. (1999). Functional attributes discriminating mechano-insensitive and mechano-responsive C nociceptors in human skin. J. Neurosci. 19, 10184–10190. 10559426 10.1523/JNEUROSCI.19-22-10184.1999 PMC6782981

[B59] Weidner C. Schmidt R. Schmelz M. Torebjörk H. Handwerker H. (2003). Action potential conduction in the terminal arborisation of nociceptive C-fibre afferents. J. Physiol. 547, 931–940. 10.1113/jphysiol.2002.028712 12576502 PMC2342739

[B60] Wu Y. Cui Y. Huynh L. Barrelet C. J. Bell D. C. Lieber C. M. (2004). Controlled growth and structures of molecular-scale silicon nanowires. Nano Lett. 4, 433–436. 10.1021/nl035162i

[B61] Zeck G. Lambacher A. Fromherz P. (2011). Axonal transmission in the retina introduces a small dispersion of relative timing in the ganglion cell population response. PLoS ONE 6:e20810. 10.1371/journal.pone.0020810 21674067 PMC3107248

