# Supplementary material for: Cortical Axons, Isolated in Channels, Display Activity-Dependent Signal Modulation as a Result of Targeted Stimulation
Source: Front Neurosci. 2016 Mar 7;10:83. doi: 10.3389/fnins.2016.00083 (PMC4779934; doi:10.3389/fnins.2016.00083)
Supplement: Supplementary file 1 [file DataSheet1.DOCX]

***Supplementary Material***

**Cortical axons, isolated in channels, display activity-dependent signal modulation as a result of targeted stimulation**

Marta K Lewandowska^*^, Miloš Radivojević, David Jäckel, Jan Müller, and Andreas Hierlemann

*** Correspondence:** Corresponding Author: [marta.lewandowska@neuro.uu.se](mailto:marta.lewandowska@neuro.uu.se)

1. **Supplementary Figures and Tables**

## Supplementary Figures

Supplementary Figure 1. Design and layout of the poly(dimethylsiloxane) (PDMS) device on the high-density microelectrode array chip. (A) The packaged chip with the PDMS channel device on it. (B) Zoomed in view of the PDMS channel device on the chip. The two culture chambers are pill-shaped and connected with one another via thin channels through which only axons can grow. Scale bar is 1 mm. (C) Schematic of the PDMS device: two chambers (top and bottom) connected by thin channels. The grey box represents the size of the microelectrode array (11,011 Pt electrodes), which is 1.75 × 2.0 mm^2^.

Supplementary Figure 2. The same data that was shown in Figure 3D, except plotted against time rather than stimulation number. The large dip in spike height and peak in latency happens after a given time rather than after a certain number of stimulations: the curves fall on top of one another. The potential for recovery, although delayed at higher frequencies, is apparent even at 70 Hz.
