# Supplementary figures and images for: Cortical Axons, Isolated in Channels, Display Activity-Dependent Signal Modulation as a Result of Targeted Stimulation
Source: Front Neurosci. 2016 Mar 7;10:83. doi: 10.3389/fnins.2016.00083 (PMC4779934; doi:10.3389/fnins.2016.00083)

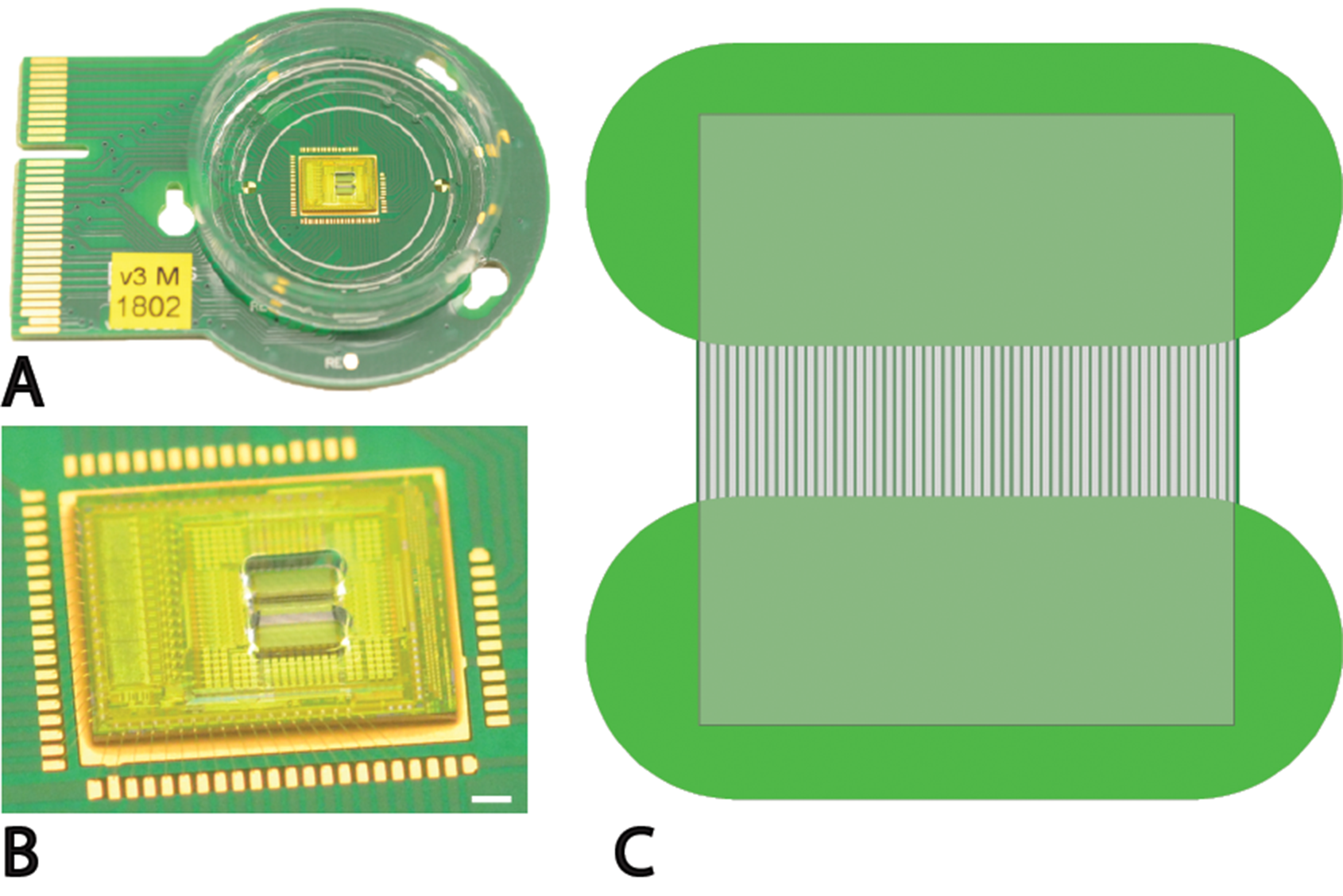

Supplement: Supplementary file 2 [file Image1.TIF]

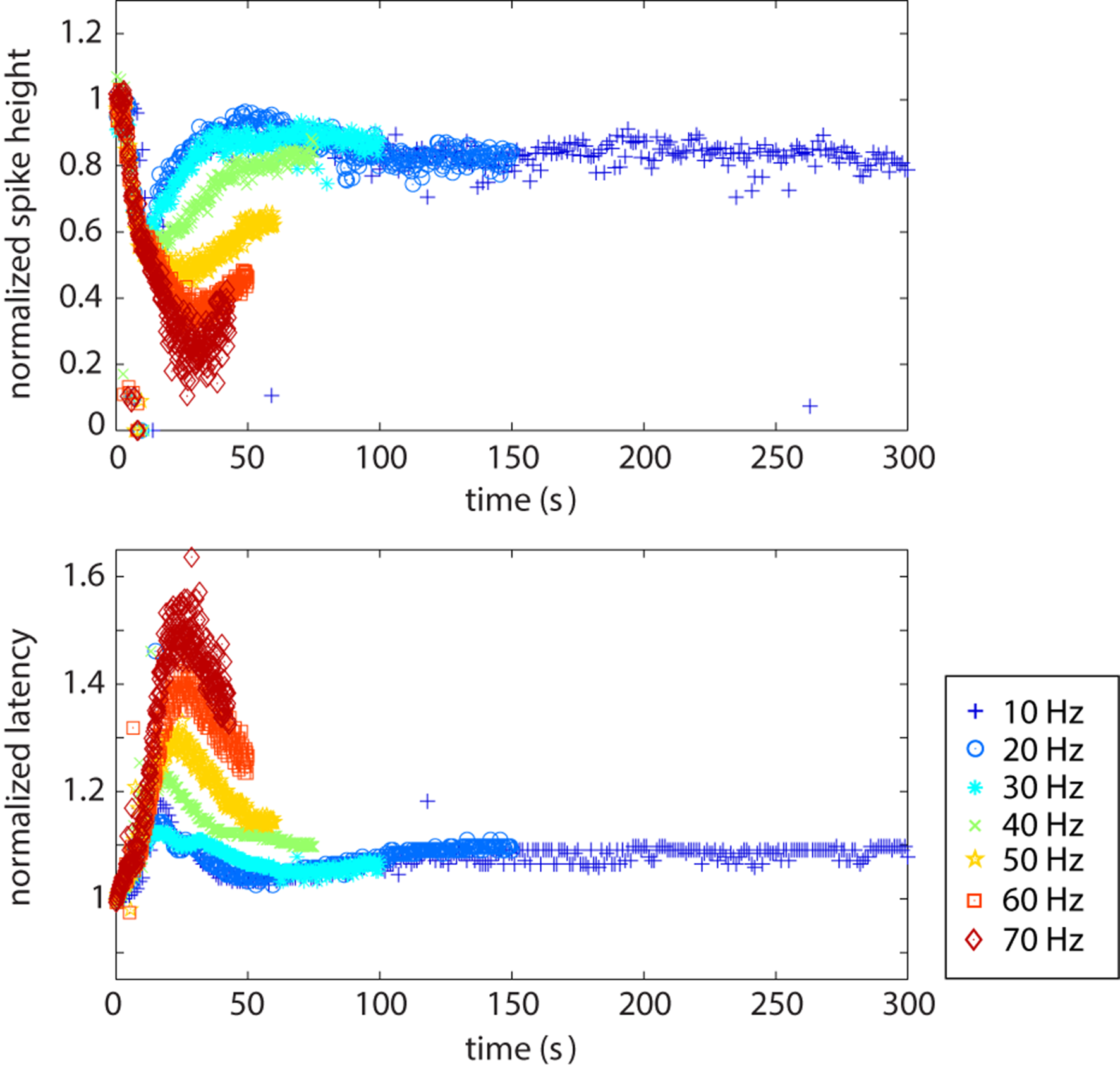

Supplement: Supplementary file 3 [file Image2.tif]
